# *UGT2B28* genomic variation is associated with hepatitis B e-antigen seroconversion in response to antiviral therapy

**DOI:** 10.1038/srep34088

**Published:** 2016-09-26

**Authors:** Kung-Hao Liang, Chih-Lang Lin, Chao-Wei Hsu, Ming-Wei Lai, Rong-Nan Chien, Chau-Ting Yeh

**Affiliations:** 1 Liver Research Center, Chang Gung Memorial Hospital, Taoyuan, Taiwan; 2 Molecular Medicine Research Center, Chang Gung University, Taoyuan, Taiwan; 3 Liver Research Unit, Keelung Chang Gung Memorial Hospital, Keelung, Taiwan; 4 Chang Gung University, College of Medicine, Taiwan

## Abstract

Seroconversion of hepatitis B virus (HBV) e-antigen (HBeAg) is a critical but often-missed therapeutic goal in standard antiviral treatments. An extreme-phenotype genome-wide association study was performed, comparing untreated spontaneous recoverers (with seroconversion of HBV surface antigen) versus entecavir-treated patients failing to achieve HBeAg seroconversion. A single-nucleotide-polymorphism rs2132039 on the *UGT2B28* gene, alongside an adjacent copy number polymorphism (CNP605), manifested the strongest clinical associations (P = 3.4 × 10^−8^ and 0.001, respectively). Multivariate analysis showed that rs2132039-TT genotypes, but not CNP605 copy numbers, remained associated to spontaneous recoverers (P = 0.009). The clinical association of rs2132039 was validated successfully in an independent cohort (n = 302; P = 0.002). Longitudinal case-only analyses revealed that the rs2132039-TT genotype predicted shorter time-to-HBeAg-seroconversion in all antiviral-treated patients (n = 380, P = 0.012), as well as the peginterferon-treated subgroup (n = 123; P = 0.024, Hazard ratio [HR] = 2.104, Confidence interval [CI] = 1.105–4.007). In the entecavir-treated subgroup, the predictive effect was restricted by pretreatment alanine aminotransferase (ALT) and aspartate aminotransferase (AST) levels, with effective prediction observed in patients with ALT < 200 IU/ml and ALT/AST ratio <2 (n = 132; P = 0.013, HR = 10.538, CI = 1.420–78.196).

Hepatitis B virus (HBV) infects more than one fourth of the global population^1^. While many infected patients recovered spontaneously, others become chronic carriers, with an estimation of 350 million worldwide, tested positive for serum HBV surface antigen (HBsAg)^1^. In the natural history of chronic hepatitis B, four distinct clinical phases have been recognized: the immune tolerant phase, the immune clearance phase, the inactive residual phase, and the reactivation phase^2^. Patients in the immune tolerant phase have serologically detectable HBV e-antigen (HBeAg) and high levels of viral DNA, albeit no apparent signs of hepatitis activities. When immune clearance begins, battles between host immunological system and HBV inevitably damage the liver, resulting in hepatonecroinflammation and the release of aspartate transaminase (AST) and alanine transaminase (ALT) into the circulation. In the meantime, the viral load gradually reduces, leading to disappearance of serum HBeAg and the appearance of anti-HBeAg antibody, namely HBeAg seroconversion. Following this event, the inactive residual phase is established. It is believed that the host immunity at this stage, signatured by positive anti-HBeAg antibody, could keep HBV replication under control^2^. To avoid liver damage, but retain the benefit of HBeAg seroconversion, current therapeutic recommendations in international guidelines suggest therapeutic interventions for patients in the immune clearance phase^3^,^4^,^5^. Accumulated evidence supported the view that antiviral therapy effectively prevented severe hepatitis flares and reduced the risks of subsequent sequels, such as decompensated liver cirrhosis and hepatocellular carcinoma (HCC)^6^,^7^,^8^, particularly in patients who achieved HBeAg seroconversion^9^. As such, achievement of HBeAg seroconversion became an essential goal for antiviral therapy^3^,^4^,^5^,^9^.

Currently approved anti-HBV drugs include immune modulators (conventional and pegylated interferon (peginterferon)) and direct antiviral nucleos(t)ide analogs (including lamivudine, entecavir, telbivudine, adefovir and tenofovir). These drugs facilitate the patients to achieve viral load reduction, ALT normalization, liver histological improvement, and HBeAg seroconversion in HBeAg-positive patients^10^,^11^,^12^. According to the results of phase III clinical trials, HBeAg seroconversion rates are 32% at week 72 in peginterferon-treated patients^11^, 31% at week 96 in entecavir-treated patients^10^, and 21% at week 48 in tenofovir-treated patients^12^. Post-marketing real-world data showed that 47–85% patients cannot achieve HBeAg seroconversion even after long-term use of nucleos(t)ide analogs^13^.

Despite the obvious person-to-person clinical variation in HBeAg seroconversion, its underlying causes remain largely unexplained. To address this issue, we searched for associated genomic biomarkers, first by a cross-sectional genome-wide association study (GWAS) with independent cohort validation, followed by longitudinal case-only analyses of patients treated by peginterferon or entecavir, the first-line immune modulator and nucleos(t)ide analog respectively in Taiwan. The GWAS was designed based on the “extreme-phenotype” strategy which has been proved effective in other genome-wide studies^14^,^15^,^16^.

## Patients and Methods

### Study subjects

This study was conducted under approval of the institutional review board, Chang Gung Memorial Hospital, Taiwan. It was carried out in accordance of the Declaration of Helsinki ethical principles on human studies. All subjects were Asian who were either examined or treated in the Linko or Keelung Medical Centers of Chang Gung Memorial Hospital. All of them have given written informed consent. The extreme-phenotype GWAS was performed on 27 unrelated patients who did not achieve HBeAg-seroclearance after two years of continuous entecavir treatment (referred to as the delayed converters), and 30 spontaneous recoverers who were negative for serum HBsAg but positive for anti-HBsAg and anti-hepatitis B core antigen (HBcAg) antibodies, an evidence of spontaneous recovery from past HBV infection. These patients had never received any antiviral therapy. Supposedly, HBV was cleared completely by these patients’ own immune systems.

Subsequent validation and longitudinal studies were performed on 110 spontaneous recoverers, 326 carriers in the inactive residual clinical phase (post HBeAg seroconversion, normal ALT), and 380 patients in the immune-clearance phase who were treated between years 2001 and 2013. The patients were treated by entecavir (n = 257) or peginterferon (n = 123) respectively according to international guidelines. Patients’ pretreatment clinical data, including age, gender, AST, ALT, HBV DNA, platelet count, and cirrhosis status were retrieved from clinical records. Taken together, a total of 873 subjects were analyzed in this study.

### DNA extraction from clinical samples

Genomic DNA was extracted from patients’ peripheral blood cells (for GWAS) or serum samples (for validation and case-only analyses) stored in the serum bank of Liver Research Center, Chang Gung Memorial Hospital, using the QIAamp DNA Mini and Blood Mini kits (Qiagen, Düsseldorf, Germany).

### Genome-wide variants calling and analysis

GWAS was performed using the Affymetrix SNP 6.0 array (Affymetrix, Santa Clara, CA), which detected genotypes of single nucleotide polymorphisms (SNP) and copy number polymorphisms (CNP) by use of the probe-target hybridization method with fluorescent signaling. SNP base calling and CNP calling were performed by the Genotyping Console™ software (Affymetrix, Santa Clara, CA) using the Birdseed and the Canary algorithms respectively. The forced-call option was not selected for SNPs calling, allowing some bases un-called if the fluorescent signal could not support a clear-cut base call. The percentage of called bases (i.e. base-call rates) was then employed as the standard for quality assurance, where SNPs with <90% base-calling rates were removed from analysis. Univatiate genome-wide associations were analyzed by the logistic-regression based allelic test using the SNP & Variation Suite (Golden Helix, Bozeman, MT).

### Focused genotyping of rs2132039

Conventional Sanger sequencing was performed to the surrounding region of rs2132039, amplified by polymerase chain reaction (PCR) with the primers 5-GAGGCTCCATCATAGTCTGGC-3′ and 5′-TTGCCTGGCTTCTCATTGTT-3′. Base-calling was performed on the sequencing trace files using novoSNP, a bioinformatics freeware^17^. Ambiguous bases which could not be recognized by the software were manually called by human curators.

### Statistical analysis

Logistic regression was used for the univariate and multivariate cross-sectional analysis. Variables which showed significant association (defined as P < 0.05) were entered into the multivariable analysis. Cox proportional hazards models were used for the univariate and multivariate longitudinal analyses on the time-to-HBeAg seroconversion. Cumulative incidences of HBeAg seroconversion in patient groups stratified by the rs2132039 genotypes were depicted using the Kaplan-Meier plots and compared by log-rank tests. All the statistic tests were two-tailed.

## Results

### Single nucleotide and copy number polymorphisms identified in GWAS

**Figure 1:**
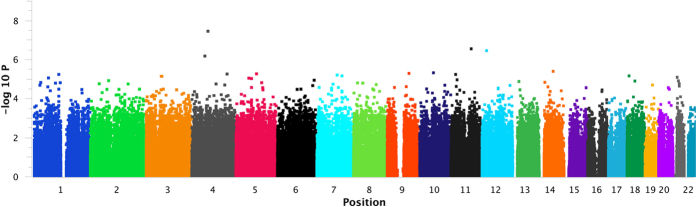
The Manhattan plot to illustrate the signal of association across all autosomal chromosomes. Horizontal axis indicated the chromosomal locations. Vertical axis indicated the P values in the negative base-10 logarithmic scale.

The extreme-phenotype GWAS was performed by comparing two groups of individuals with extreme phenotypes: delayed converters (for HBeAg seroconversion) and spontaneous recoverers (see Patients and Methods section for patient definition). No significant differences of age and gender distributions were found between the two groups (Table 1). A total of 804,491 single nucleotide polymorphisms (SNP) across the 22 autosomal chromosomes reached the quality standard. Among them, 478,372 SNPs had minor allele frequency ≧10% and were included for statistical analysis without any computational imputations. A peak signal of association was found in the chromosome 4q13.2 region (Fig. 1, logistic-regression based allelic test P = 3.4 × 10^−8^, after Bonforroni correction, P = 0.0165). The inflation factor^18^ was 1.0808. This peak signal occurred at the genomic variant rs2132039 (alleles: T/C) in the intronic region of the UDP Glucuronosyltransferase 2 family, polypeptide B28 (*UGT2B28*) gene, where the allele “T” was associated to spontaneous recoverers. Additionally, an adjacent copy number polymorphism CNP605 with an annotated length of 111,456 bases (alleles: Presence/Absence of the DNA segment) was also identified, where the presence of the DNA segment was associated to spontaneous recoverers (Table 1, P = 0.001). The SNP-calls and the CNP-calls were highly correlated in the way that rs2132039-TT was associated to CNP605-2-copies, while the non-TT (including TC and CC) was associated to the reduced CNP605 copy number (Fisher’s exact P = 4.1 × 10^−8^), achieving a sensitivity of 100% and a specificity of 74.19%. When both the SNP and CNP genotypes were entered into the multivariate logistic regression analysis of clinical associations, only SNP genotype remained statistically significant (Table 1, adjusted P = 0.009). Therefore, we selected the SNP for subsequent validation study.

### Cross-sectional validation of the GWAS discovery

Independent cohorts of 110 spontaneous recoverers, 326 carriers in the inactive-residual phase (HBsAg positive, HBeAg negative, anti-HBe positive and normal ALT), and 380 HBeAg-positive patients in the immune clearance phase were then recruited. Of patients in the immune clearance phase, we identified 192 patients who had received entecavir treatment continuously but failed to achieve HBeAg seroconversion after 2 years of therapy. They were identified as the “delayed converters”, and their rs2132039 genotypes were compared with the 110 spontaneous recoverers for a cross-sectional validation. Age and genotype distributions of the two groups showed significant difference in the univariate analysis (Table 2, P < 0.001 and = 0.001 respectively). After adjusted for age, the rs2132039 genotype remained statistically significant (Table 2, adjusted P = 0.002, Odds ratio = 2.584, confidence interval = 1.424–4.690). The genotype distribution of subjects in the inactive-residual phase also deviated significantly from that of the spontaneous recoverers (Table 2, P = 0.001).

A significant difference of *UGT2B28* genotype distribution was also found between patients with or without HBeAg seroconversion 200 days after the start of entecavir treatment (Fisher’s exact test, P = 0.033, N = 257).

### The “TT” genotype and peginterferon treatment independently predict shorter time-to-HBeAg-seroconversion

**Figure 2:**
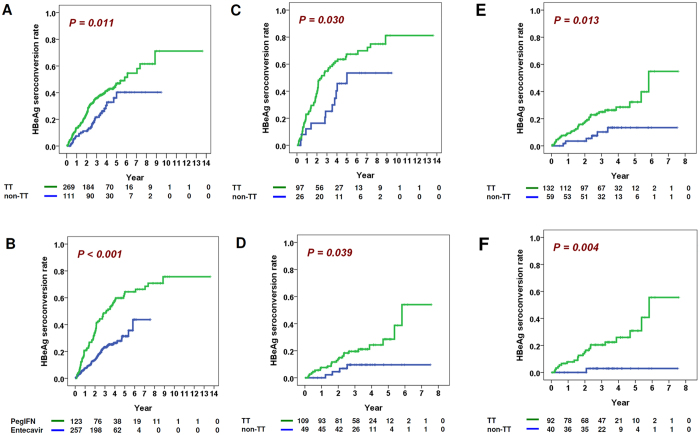
Cumulative HBeAg seroconversion rates of 380 immune-clearance patients. (**A**) patients stratified by the “TT” and “non-TT” genotypes; (**B**) patients stratified by treatment methods: peginterferon versus entecavir; (**C**) peginterferon-treated patients stratified by the “TT” and “non-TT” genotypes; (**D**) entecavir-treated patients with pretreatment ALT < 200, stratified by the “TT” and “non-TT” genotypes; (**E**) entecavir-treated patients with ALT/AST < 2, stratified by the “TT” and “non-TT” genotypes; (**F**) entecavir-treated patients with ALT < 200 and ALT/AST < 2, stratified by the “TT” and “non-TT” genotypes. All the P values were derived from log-rank tests.

Longitudinal analysis was then performed to understand the dynamic clinical outcome of the immune-clearance patients. Five factors, including age (P = 0.002), cirrhosis (P = 0.003), ALT/AST ratio (P = 0.034), rs2132039 genotype (P = 0.012) and treatment methods (P < 0.001), were significantly associated to time-to-HBeAg seroconversion in the univariate analysis (Table 3). When these factors were incorporated into a multivariate analysis, only rs2132039 and treatment methods remained significantly associated (Table 3, adjusted P = 0.046 and < 0.001 respectively). Higher cumulative HBeAg seroconversion rates were found on the TT-type patients than the non-TT patients (Fig. 2A, Log-Rank P = 0.011). Also, higher cumulative HBeAg seroconversion rates were observed in patients treated by peginterferon than by entecavir (Fig. 2A, Log-Rank P < 0.001). We then investigated the role of rs2132039 genotypes in the two treatment subgroups separately. In peginterferon-treated patients, AST (P = 0.012), ALT (P = 0.003) and rs2132039 genotypes (P = 0.033) were significantly associated to time-to-HBeAg seroconversion in the univariate analysis (Table 4). Only rs2132039 genotypes remained significantly associated in the multivariate analysis (P = 0.024, adjusted Hazard ratio = 2.104, Confidence interval = 1.105–4.007). Patients with the TT type had higher cumulative HBeAg seroconversion rates than those with the non-TT type (Log-Rank P = 0.030, Fig. 2C).

**Figure 3:**
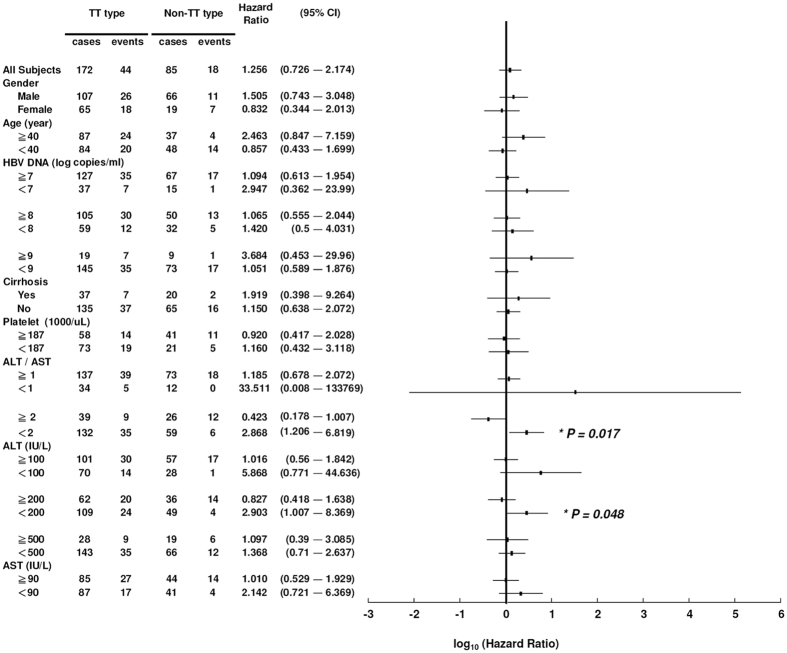
The forest plot to illustrate hazard ratios and confidence intervals of time-to-HBeAg seroconversion of “TT” versus “Non-TT” patients in various clinical strata. Significant associations were found in the ALT/AST < 2 and ALT < 200 strata.

In the entecavir-treatment subgroup, no clinical factors were found to be associated to HBeAg seroconversion (Supplementary Table S1). However, among various patient strata, the rs2132039 genotype was associated to HBeAg seroconversion when ALT < 200 IU/ml or ALT/AST < 2 (Fig. 3, Cox P = 0.048 and 0.017 respectively). In the two strata, patients with the TT type had higher cumulative HBeAg seroconversion rates than those with the non-TT type (log-Rank P = 0.039 and 0.013 respectively, Fig. 2D,E). For patients fulfilling both criteria at the same time, more prominent differences of cumulative HBeAg seroconversion rates were observed (log-Rank P = 0.004, Cox P = 0.021, Fig. 2F), achieving a large hazard ratio of 10.538 (Confidence interval = 1.420–78.196). These results indicated that in the entecavir-treated patients, the rs2132039 genotype predictor was only effective when patients had not experienced pre-therapeutic hepatitis flares. In other words, pre-therapeutic hepatitis activities (ALT > 200 IU/L or ALT/AST > 2) abolished this correlation.

In this longitudinal cohort, the HBV genotypes in 320 patients were assessed retrospectively using stored serum samples, whereas in the remaining 60 patients, the HBV genotypes were unknown due to lack of samples. Of them, 186 (58.1%) were genotype B, and 134 (41.9%) were genotype C. The viral genotypes (C versus B) were not significantly associated to the time-to-HBeAg seroconversion (Hazard ratio = 0.791, Confidence interval = 0.547–1.146, P = 0.215). However, when patients were stratified by the HBV genotypes B or C, the cumulative HBeAg seroconversion rates were associated to *UGT2B28* genotypes only in viral genotype B (Supplementary Fig. 1A, log-rank P = 0.037, “TT” versus “non-TT” Hazard ratio = 1.872, Confidence interval = 1.027–3.412) but not in viral genotype C (Supplementary Fig. 1B, log-rank P = 0.848, Hazard ratio = 1.067, Confidence interval = 0.548–2.080). Treatment subgroup analysis showed that the time-to-HBeAg seroconversion was not significantly associated to the viral genotypes in either the peginterferon-treated patient stratum (Hazard ratio = 0.886, Confidence interval = 0.541–1.450, P = 0.629, N = 99) or the entecavir-treated patient stratum (Hazard ratio = 0.706, Confidence interval = 0.403–1.239, P = 0.225, N = 221).

## Discussion

In patients with chronic HBV infection, hepatitis activities could perpetuate for decades, if not a lifetime. During this long period of time, the disease can progress into various distinct clinical outcomes, owing to the complex and dynamic viral-host interactions as well as the significant variations in therapeutic responses to antiviral therapy. In this study, we asked whether the genetic background played a role in determining these different clinical/therapeutic outcomes. The extreme-phenotype GWAS approach was adopted because of the presence of a great range of clinical outcomes. We first investigated whether there was a genetic marker capable of distinguishing the two groups of patients with extreme phenotypes (spontaneous recoverers versus delayed converters). Then, patients with other clinical phenotypes in-between were analyzed for correlations. It was discovered that the *UGT2B28* genotype not only correlated with the two extreme phenotypic groups but also served as a predictor for HBeAg seroconversion in patients receiving two different kinds of antiviral therapies.

Overall, the multivariate analysis showed that the “TT” genotype and peginterferon treatment were independent predictors for shorter time-to-HBeAg-seroconversion. Subgroup analysis revealed that the “TT” genotype remained in association with shorter time-to-HBeAg-seroconversion in peginterferon-treated patients. Nevertheless, in entecavir-treated patients, we found an interesting interfering role of ALT levels in the effectiveness of *UGT2B28* genotype as a predictor for HBeAg seroconversion. Severe ALT flares have been reported as a strong affecter for HBeAg seroconversion^19^. It can be deduced that ALT flares are the consequence of hepatic necroinflammation caused by host immunological activity, which leads to viral load reduction and, if great enough, results in HBeAg clearance and production of anti-HBeAg antibodies (HBeAg seroconversion). In entecavir-treated patients, genotype association was only found in patients with less significant pretreatment flares, for example, ALT < 200 or ALT/AST < 2. In contrast, the genotype remained significantly associated with seroconversion in peginterferon-treated patients even after adjustment for AST and ALT levels. The reason for such distinction between different antiviral treatments was unknown. We speculate that because peginterferon is an immune modulator, which utilizes immune-mediated mechanisms similar to those of ALT flares to clear HBV viral load, therefore, the predictive effect of *UGT2B28* genotype in this cohort of patients is not affected by pre-treatment ALT levels. On the other hand, entecavir is a viral polymerase inhibitor, which suppresses HBV replication without affecting significantly the host immune system. As a result, the predictive effect of genotype on HBeAg seroconversion was conditioned on the host immunological status partly represented by pre-treatment ALT levels.

One novel finding of this GWAS was the co-identification of adjacent single nucleotide and copy number polymorphisms as significant association factors. In our data, 66.67% of the spontaneous recoverers have the 2-copy genotype, while 33.33% have the 1-copy genotype. This is similar to the HapMap Chinese + Japanese cohort (n = 89), where the percentages of subjects with the 2-copy, the 1-copy and the completely deleted (i.e. 0-copy) genotypes are 71.9%, 25.8% and 2.2% respectively (Cochran-Armitage Trend test P = 0.776)^20^. CNP605 was annotated at the position of 70,162,233-70,273,689 bases of chromosome 4 in the GRCh37 version of the human genome^20^. Genomic sequences surrounding CNP605 manifested a historical non-allelic homologous recombination, characterized by a segmental duplication in tandem and a reduced copy number in between^21^,^22^,^23^,^24^,^25^,^26^,^27^. Apart from *UGT2B28* and one paralogous pseudogene *UGT2B24P*^28^, no other genes/pseudogenes were annotated in this region. We hypothesized that the structural polymorphism of CNP605 (copy number difference) may contribute to alteration of *UGT2B28* expression levels, and thus carrying mechanistic implications.

The *UGT2B28* protein is a phase II xenobiotic metabolizing enzyme in the liver. It is known for its capability of transferring glucuronic acid from uridine diphosphoglucuronic acid to a diverse range of substrates including steroid hormones and lipid-soluble drugs^29^. The glucuronic substrates are more water soluble and excretable through renal route^30^. The known substrates include 5-beta-androstane 3-alpha, 17-beta-diol, estradiol, androsterone, eugenol and bile acids^31^,^32^. Of them, sex hormones have been extensively studied for their capability of regulating HBV replication^33^. Presumably, alteration of *UGT2B28* function could affect metabolism of sex hormones and thus interfere with HBV replication^32^. Another less recognized function of the UGT2B gene family is their capability to serve as a human minor histocompatibility antigen. At this time, the major clinical role in this aspect is their contributions in organ transplantation. For example, mismatch of UGT2B17 expression could lead to increased risk of graft-versus-host disease^34^. Whether this putative histocompatibility antigen contributes to immune mediated HBV viral clearance deserves further investigation.

In conclusion, we found the *UGT2B28*-rs2132039 genotypes were associated with time-to-HBeAg-seroconversion upon peginterferon or entecavir treatments in chronic hepatitis B.

## Additional Information

**How to cite this article**: Liang, K.-H. *et al*. *UGT2B28* genomic variation is associated with hepatitis B e-antigen seroconversion in response to antiviral therapy. *Sci. Rep.* **6**, 34088; doi: 10.1038/srep34088 (2016).

## Supplementary Material

Supplementary Information

## Figures and Tables

**Table 1:** Analysis of the exploratory cross-sectional cohort.

	Study Groups		Univariate Analysis		Multivariate Analysis	
	Spontaneous recoverer	Delayed HBeAg converter	Odds Ratio (95% Confidence Interval)	P	Odds Ratio (95% Confidence Interval)	P
Subject number	30	27				
Age (year)	44.63 ± 5.83	45.63 ± 9.72	0.984 (0.920–1.052)	0.630		
Gender-Male (%)	20 (66.7%)	20 (74.07%)	0.700 (0.222–2.206)	0.542		
SNP rs2132039 TT (%)*	24 (82.76%)	7 (30.43%)	11.429 (3.303–39.549)	**<0.001**	6.934 (1.626–29.568)	**0.009**
non-TT (%)	5 (17.24%)	16 (69.57%)				
CNP605- 2 copies (%)*	20 (66.67%)	6 (22.22%)	7.000 (2.145–22.848)	**0.001**	2.449 (0.567–10.585)	0.230
1 copy (%)	10 (33.33%)	21 (77.78%)				

*Subjects with ambiguous basecalls or CNP calls by the Birdseed v2 and Canary algorithms are not counted

**Table 2:** Cross-sectional validation analysis.

	Study Groups		Univariate Analysis		Multivariate Analysis	
	Spontaneous recoverer	Delayed HBeAg converter	Odds Ratio (95% Confidence Interval)	P	Odds Ratio (95% Confidence Interval)	P
Subject number	110	192				
Age (year)	49.93 ± 14.62	43.58 ± 11.61	1.038 (1.019–1.058)	**<0.001**	1.036 (1.016–1.056)	**<0.001**
Gender-Male (%)	63 (57.3%)	129 (67.19%)	0.655 (0.404–1.061)	0.086		
SNP rs2132039 TT (%)	92 (83.64%)	124 (64.58%)	2.803 (1.561–5.033)	**0.001**	2.584 (1.424–4.690)	**0.002**
	**Spontaneous recoverer**	**Inactive-residual subjects**				
Subject number	110	326				
Age (year)	49.93 ± 14.62	50.92 ± 11.59	0.994 (0.976–1.011)	0.470		
Gender-Male (%)	63 (57.3%)	194 (59.51%)	0.912 (0.589–1.413)	0.680		
SNP rs2132039 TT (%)	92 (83.64%)	219 (67.18%)	2.497 (1.433–4.352)	**0.001**	2.497 (1.433–4.352)	**0.001**

**Table 3:** Cox regression analysis of the time-to-HBeAg seroconversion in patients in the immune-clearance phase.

	Baseline Statistics	Univariate			Multivariate		
		Hazard Ratio	(95% CI)	P	Hazard Ratio	(95% CI)	P
Subject number	380						
Gender-Male (%)	258 (67.89%)	0.781	(0.551–1.106)	0.164			
Age (year)	40.27 ± 11.73	0.975	(0.96–0.991)	**0.002**	0.985	(0.969–1.001)	0.064
HBV DNA (log copies/ml)	7.98 ± 1.42	1.012	(0.895–1.143)	0.854			
Cirrhosis (%)	66 (17.37%)	0.409	(0.226–0.74)	**0.003**	0.570	(0.305–1.065)	0.078
platelet (1000/uL)	194.08 ± 76.03	1.002	(1–1.003)	0.091			
ALT/AST	1.65 ± 0.63	1.329	(1.021–1.729)	**0.034**	1.017	(0.753–1.374)	0.912
ALT (IU/L)	286.59 ± 410.97	1.000	(1–1.001)	0.243			
AST (IU/L)	179.88 ± 276.57	1.000	(1–1.001)	0.567			
SNP rs2132039 TT (%)	269 (70.79%)	1.691	(1.122–2.548)	**0.012**	1.523	(1.007–2.302)	**0.046**
Peginterferon treatment	123 (32.37%)	2.563	(1.82–3.608)	**<0.001**	2.197	(1.547–3.121)	**<0.001**

CI, confidence interval.

**Table 4:** Cox regression analysis of the peginterferon-treated patient group with respect to the time to HBeAg seroconversion.

	Baseline Statistics	Univariate			Multivariate		
		Hazard Ratio	(95% CI)	P	Hazard Ratio	(95% CI)	P
Subject number	123						
Gender-Male (%)	85 (69.11%)	0.889	(0.549–1.438)	0.631			
Age (year)	37.84 ± 11.2	0.983	(0.963–1.003)	0.094			
HBV DNA (log copies/ml)	7.87 ± 1.68	0.980	(0.854–1.124)	0.774			
Cirrhosis (%)	9 (7.32%)	0.413	(0.129–1.318)	0.135			
platelet (1000/uL)	201.95 ± 83.64	1.001	(0.999–1.003)	0.305			
ALT/AST	1.8 ± 0.56	1.040	(0.694–1.559)	0.849			
ALT (IU/L)	194.87 ± 169.18	1.001	(1–1.002)	**0.012**	1.000	(0.998–1.002)	0.832
AST (IU/L)	122.67 ± 93.04	1.003	(1.001–1.005)	**0.003**	1.004	(0.999–1.008)	0.111
SNP rs2132039 TT (%)	97 (78.86%)	2.007	(1.057–3.813)	**0.033**	2.104	(1.105–4.007)	**0.024**

CI, confidence interval.
