# Supplementary material for: UGT2B28 genomic variation is associated with hepatitis B e-antigen seroconversion in response to antiviral therapy
Source: Sci Rep. 2016 Sep 26;6:34088. doi: 10.1038/srep34088 (PMC5036189; doi:10.1038/srep34088)
Supplement: Supplementary Information [file srep34088-s1.pdf]

UGT2B28 genomic variation is associated with hepatitis B e-antigen seroconversion in response to antiviral therapy

Kung-Hao Liang[1,3#], Chih-Lang Lin[1,2,4#], Chao-Wei Hsu[1,4], Ming-Wei Lai[1,4], Rong-Nan Chien[2], Chau-Ting Yeh[1,3,4*]

#These two authors contributed equally

Affiliations

[1] Liver Research Center, Chang Gung Memorial Hospital, Taoyuan, Taiwan

[2] Liver Research Unit, Keelung Chang Gung Memorial Hospital, Keelung, Taiwan

[3] Molecular Medicine Research Center, Chang Gung University, Taoyuan, Taiwan

[4] Chang Gung University, College of Medicine, Taiwan

Supplementary Table S1: Cox regression analysis of the entecavir-treated patient group with respect to time-to-HBeAg seroconversion

	Baseline Statistics	Univariate		
		Hazard Ratio	(95% CI)	P
**Subject number**	257			
**Gender-Male (%)**	173 (67.32%)	0.660	(0.397-1.1)	0.111
**Age (year)**	41.41±11.83	0.980	(0.957-1.003)	0.086
**HBV DNA (log copies/ml)**	8.03±1.27	1.124	(0.905-1.396)	0.292
**Cirrhosis (%)**	57 (22.18%)	0.626	(0.333-1.176)	0.145
**platelet (1000/uL)**	189.1±70.58	1.001	(0.997-1.005)	0.534
**ALT/AST**	1.59±0.64	1.337	(0.921-1.939)	0.127
**ALT (IU/L)**	212.04±325.44	1.000	(1-1.001)	0.105
**AST (IU/L)**	330.67±480.27	1.000	(1-1.001)	0.250
**SNP rs2132039 TT (%)**	172 (66.93%)	1.256	(0.726-2.174)	0.415

CI, confidence interval

A

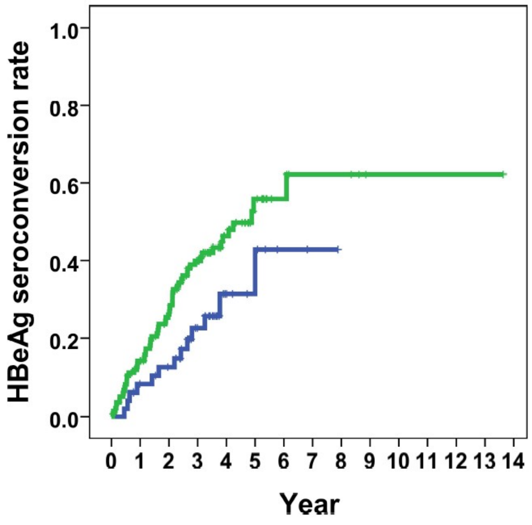

B

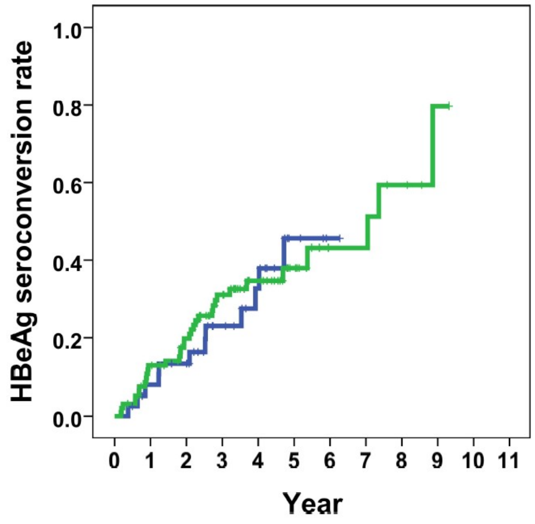

Figure S1. The Kaplan-Meier analysis curves for the patients with HBV genotype B (A) and genotype C (B). The HBeAg seroconversion rate between *UGT2B28* genotypes "TT" (green) and "non-TT" (blue) were significantly different in genotype B (log-rank P = 0.037, Hazard ratio = 1.872, Confidence interval = 1.027 – 3.412) but not in genotype C patients (log-rank P = 0.848, Hazard ratio = 1.067, Confidence interval = 0.548 – 2.080).
